# Overexpression of *Populus trichocarpa* Mitogen-Activated Protein Kinase Kinase4 Enhances Salt Tolerance in Tobacco

**DOI:** 10.3390/ijms18102090

**Published:** 2017-10-18

**Authors:** Chengjun Yang, Ruoning Wang, Luzheng Gou, Yongchao Si, Qingjie Guan

**Affiliations:** Northeast Forestry University, Harbin 150040, China; nxyycj@sina.cn (C.Y.); WRN0812@126.com (R.W.); 18746014246@163.com (L.G.); siyongchao100@126.com (Y.S.)

**Keywords:** *Populus trichocarpa*, mitogen-activated protein kinase, salt stress, tobacco, transgene

## Abstract

Mitogen-activated protein kinase (MAPK) is one of the factors of cascade reactions affecting responses to signal pathway of environmental stimuli. Throughout the life of plants, MAPK family members participate in signal transduction pathways and regulate various intracellular physiological and metabolic reactions. To gain insights into regulatory function of MAPK kinase (MAPKK) in *Populus trichocarpa* under salt stress, we obtained full-length cDNA of *PtMAPKK4* and analyzed different expression levels of *PtMAPKK4* gene in leaves, stems, and root organs. The relationship between PtMAPKK4 and salt stress was studied by detecting expression characteristics of mRNA under 150 mM NaCl stress using real-time quantitative polymerase chain reaction. The results showed that expression of *PtMAPKK4* increased under salt (NaCl) stress in leaves but initially reduced and then increased in roots. Thus, salt stress failed to induce PtMAPKK4 expression in stems. PtMAPKK4 possibly participates in regulation of plant growth and metabolism, thereby improving its salt tolerance. We used *Saccharomyces cerevisiae* strain INVScI to verify subcellular localization of PtMAPKK4 kinase. The yeast strains containing pYES2-PtMAPKK4-GFP plasmid expressed GFP fusion proteins under the induction of d-galactose, and the products were located in nucleus. These results were consistent with network prediction and confirmed location of PtMAPKK4 enzyme in the nucleus. We tested NaCl tolerance in transgenic tobacco lines overexpressing *PtMAPKK4* under the control of 35S promoter at germination stage to detect salt tolerance function of PtMAPKK4. Compared withK326 (a wild-type tobacco), lines overexpressing *PtMAPKK4* showed a certain degree of improvement in tolerance, germination, and growth. NaCl inhibited growth of overexpressed line and K326 at the seedling stage. However, statistical analysis showed longer root length, higher fresh weight, and lower MDA content in transgenic lines in comparison with that in K326.

## 1. Introduction

The plant life cycle experiences disturbances coming from different stresses, including biological and abiotic stresses, such as pathogen infection, salinity, drought and a low-temperature environment. These environmental stress factors negatively affect plant growth and development. Therefore, plants protect themselves from stress by a variety of physiological and morphological changes, which result from the identification of external signals in the interior of cells through signal transduction [1,2]. Mitogen-activated protein kinase (MAPK) is one of the cascade reaction factors for responses to signal pathways of environmental stimuli [3]. Throughout the life cycle of plants, MAPK family members mainly participate in signal transduction pathways and regulate various intracellular physiological and metabolic reactions [4]. MAPK cascades comprise three hierarchical protein kinases, namely, activated protein kinase kinases (APKKs), MAPK kinases (MAPKKs), and MAPKs. These protein kinases rapidly amplify signals and transduce extracellular signals to regulate various intracellular adaptive responses. MAPKKs and MAPKs play extensive roles in posttranslational regulation in diverse plants [5,6,7,8]. MAPK cascades play key roles in the transduction of hormone signals [9,10,11], plant cytokinesis [12], pollen development [13], and biotic and abiotic stresses [14,15,16,17]. As a model tree, *Populus trichocarpa* cultivation is vital for economies and ecosystems worldwide. However, biotic and abiotic stresses may adversely affect the growth and development of this plant. Thus, increasing attention has been paid to studying the molecular mechanisms underlying stress tolerance in poplar [8,18]. Eleven MAPKKs (PtMAPKKs) and twenty-one MAPKs (PtMAPKs) have been characterized in the genome of *Populus trichocarpa*. Most studies have focused on chromosomal localization, structure, and classification of *MAPKK* gene and its specific expression pattern in tissues and organs [7]. It has been reported that *PtMAPKK4* overexpressed transgenic populus showed a lower content of H_2_O_2_ under drought stress. The overexpression of the *PtMAPKK4* enhanced the activity of antioxidant enzyme through an up-regulation of its expression, and the reduction of reactive oxygen species (ROS) could improve the plant tolerance of stress [19]. However, no research has clarified the mechanisms underlying signal transduction pathway of PtMAPKK4 regarding salt-alkaline tolerance.

*MAPKK* and *MAPK* gene family members exhibit signal transduction in response to stress. Considering the central role of MAPKKs in the MAPK cascade reaction, we systemically analyzed the role of *PtMAPKK4* in abiotic stress (salt stress) and in the function of signal transduction, cloned *PtMAPKK4* through reverse transcription polymerase chain reaction (RT-PCR), analyzed the subcellular localization of MAPKK protein through yeast transformation, detected characteristics of *PtMAPKK4* gene in response to abiotic stress, and investigated overexpression of *PtMAPKK4* in response to stress in transgenic tobacco. This study provides insights into the tolerance gene for the molecular breeding of trees and serves as a foundation for studying the pathway of signal transduction for the MAPKK family.

## 2. Result

### 2.1. Bioinformatics Analysis of PtMAPKK4 Sequence

**Figure 1:**
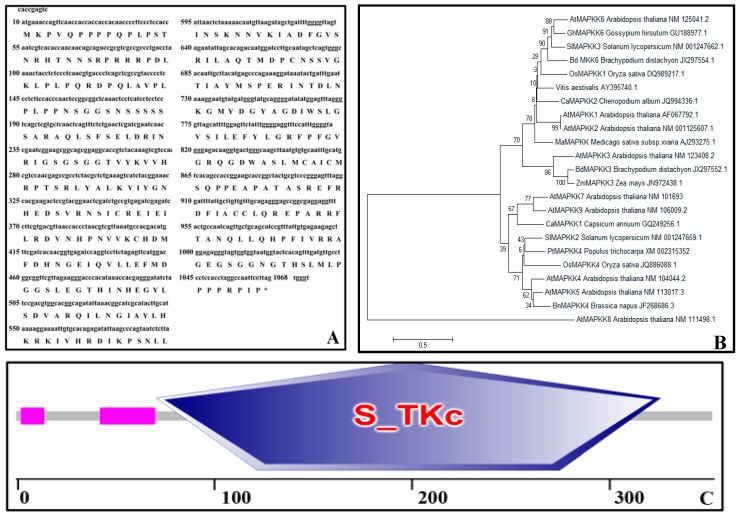
Bioinformatics analysis of PtMAPKK4 sequence. (**A**) Analysis of the open reading frame (ORF) and the coding amino acid sequence of the *PtMAPKK4* gene; (**B**) Neighbor-joining phylogenetic tree of the MAPKK gene family, as created by DNAMAN, Bootstrap value (above 50%) supports from 1000 replicates are indicated at each branch with a bootstrap value of 1000, scale is 0.05; (**C**) The S-TKc conserved domain, a sequence feature of the serine/threonine protein kinase family, was displayed by SMART.

Basic information on the *PtMAPKK4* gene was obtained from the NCBI website and used to design primers and clone full-length *PtMAPKK4* ORF, which measured 1059 bp long with 352 encoded amino acids (Figure 1A). We used the online software ExPaSy to analyze the following physicochemical protein properties: protein molecular weight (3.895 KD); isoelectric point (9.3); and grand average of hydropathicity (−0.434), which indicates the probability of hydrophobic protein identity. We researched members of the MAPKK protein family from the literature and used the software DNAMAN to construct a phylogenetic tree (Figure 1B). In this tree, PtMAPKK4 showed the highest homology with *Oryza sativa* OsMAPKK4 and *Arabidopsis* AtMAPKK4/5. It had been reported that OsMAPKK4 and AtMAPKK4 were associated with salt tolerance [20,21]. So we speculated that PtMAPKK4 might be expressed in response to salt stress.

We used the online software SMART to analyze conserved domains of PtMAPKK4 and observed that this protein features an S_TKc domain (Figure 1C), which is a sequence feature of the serine/threonine protein kinase family. Thus, we speculated that the PtMAPKK4 protein belongs to the MAPKK family.

### 2.2. PtMAPKK4 Gene Expression Properties

**Figure 2:**
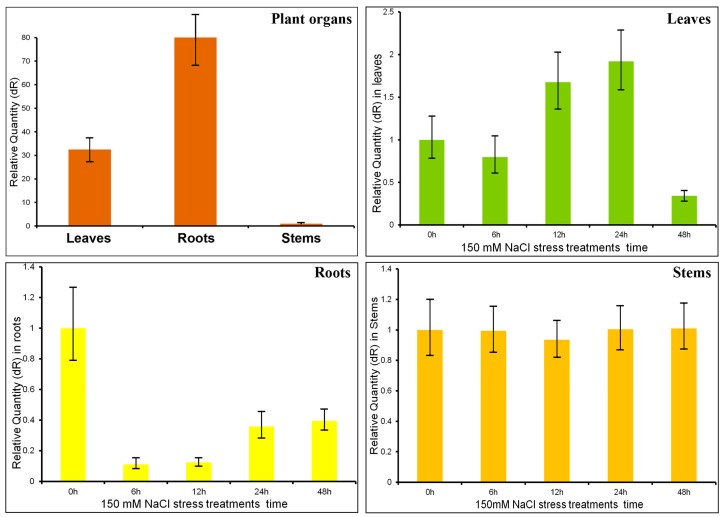
(**A**) *PtMAPKK4* transcript levels in leaves, roots, and stems and under salt stress (NaCl) in (**B**) leaves; (**C**) roots; and (**D**) stems relative to the housekeeping gene *Actin2* (error bars indicate SE of three replicates).

qRT-PCR was used to detect the expression levels of the *PtMAPKK4* gene in leaves, stems, and roots and to investigate the biological functions of the *PtMAPKK4* gene during plant growth. The lowest expression level was detected in stems, whereas the highest was measured in roots, which showed nearly the 80-times higher level than in stems (Figure 2A). Thus, the *PtMAPKK4* gene possibly mainly functions in roots and plays a role in signal amplification.

Plants regulate their physiological and biochemical metabolism to enhance their tolerance through three levels of cascade signal amplification. qRT-PCR was used to detect the expression levels of the *PtMAPKK4* gene in different tissues under 150 mM NaCl stress at different times and to reveal the correlation between the *PtMAPKK4* gene and NaCl stress. The expression level of *PtMAPKK4* in leaves increased gradually with time and reached the highest value at 24 h; this value was 1.9 times higher than that in the control. However, the expression level of *PtMAPKK4* decreased at 48 h, indicating that this gene first increased and then decreased (Figure 2B). On the other hand, the expression level in roots first decreased and then increased with time (Figure 2C). The gene expression level in stems was non-significantly changed (Figure 2D). The results showed that at the transcriptional level, the *PtMAPKK4* gene participated in salt stress in roots and leaves, that is, *PtMAPKK4* gene strongly correlated with salt stress in roots and leaves. *PtMAPKK4* possibly responded to salt stress by increasing its expression level in roots and leaves with stress time to improve salt tolerance.

### 2.3. Subcellular Localization of PtMAKK4 Protein

**Figure 3:**
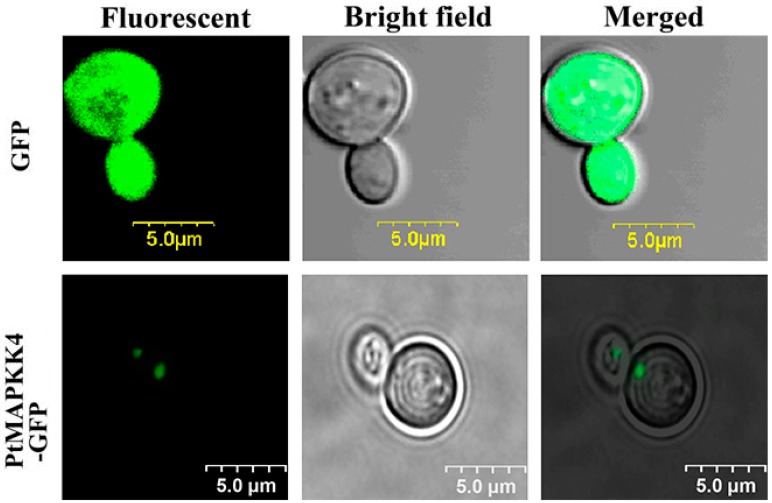
Subcellular localization of PtMAPKK4 under Fluorescent, Bright and Merged vision. GFP: green fluorescent protein.

It has been reported that plant MAPKKs are located in the nucleus [22,23]. So we wanted to verify whether the subcellular localization of PtMAPKK was same as that of other plant MAPKKs. The online software PSORT was used to predict the subcellular localization of PtMAKK4 protein. This protein exhibited a 70% probability of localization in the nucleus, 30% in the microbody, and 10% in the mitochondrial matrix. To obtain accurate information on protein subcellular localization, we continued the study using green fluorescent protein (GFP). First, we constructed the yeast expression vector pYES2*-PtMAPKK4-GFP* with a galactose inducible promoter GAL1 and GFP. Recombinant vector was transformed into *Saccharomyces cerevisiae* INVScI. After induction by d-galactose, yeast cells were imaged under fluorescence microscope (Figure 3). We detected a fluorescence signal in the nucleus only, suggesting the *PtMAPKK4* protein was localized in the nucleus. This finding agreed with the results predicted by PSORT. Therefore, the PtMAPKK4 protein functions in the nucleus.

### 2.4. Analysis of PtMAPKK4 Gene Overexpression in Response to Salt Tolerance

**Figure 4:**
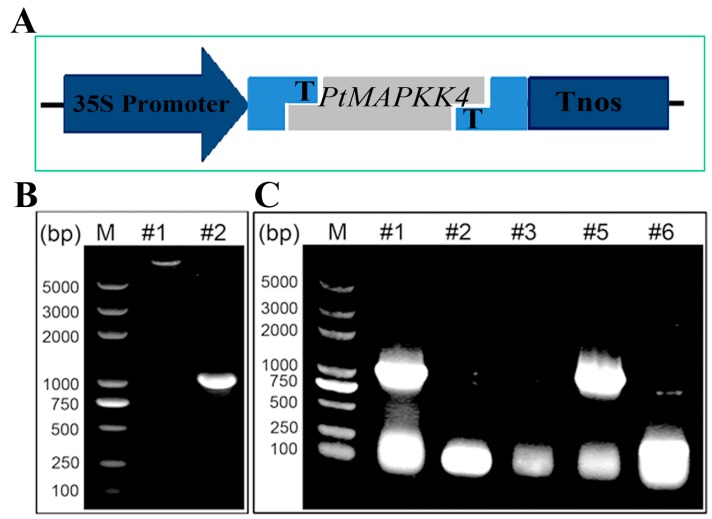
Construction of the plant expression vector *pCXSN-PtMAPKK4* plasmid. (**A**) Schematic of expression vectors *pCXSN-PTMAPKK4*; (**B**) The *PtMAPKK4* gene was amplified from cDNA of *Populus trichocarpa*, lane#1, the plasmid of plant expression vector *Pcxsn*, lane#2, amplification of the *PtMAPKK4* gene; (**C**) The *PtMAPKK4* gene was amplified from *pCXSN-PtMAPKK4* vector for verification, lane#1-6, amplification of *PtMAPKK4* in six samples, #1 and #5 were used for transgene.

**Figure 5:**
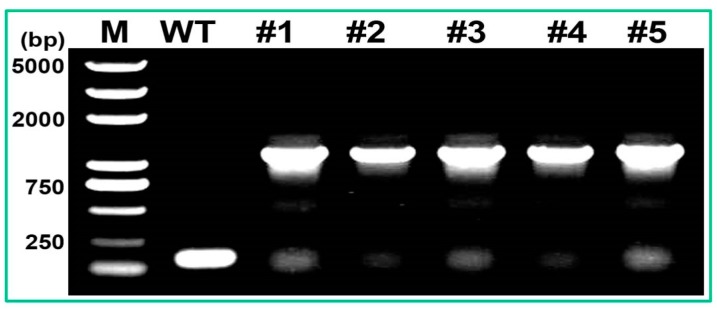
Different lines of *PtMAPKK4* transgenic tobacco as detected by PCR, M: 5kb marker; #1–5: *PtMAPKK4* of five transgenic lines.

**Figure 6:**
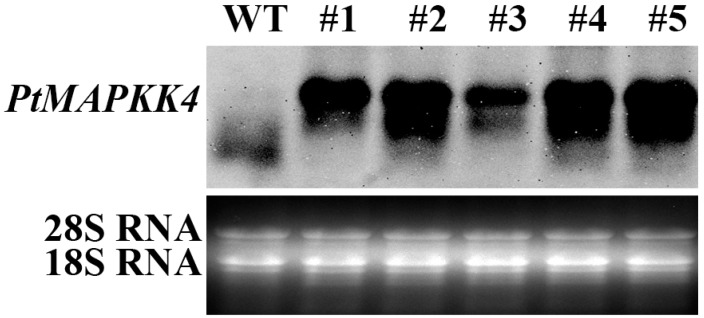
Northern blot analysis of *PtMAPKK4* expression in leaves of wild-type (WT) and transgenic lines. After isolating the total RNA, 20 µg of it was loaded in each lane. Photographs of the ethidium bromide-stained gel that was used as the loading controls.

To overexpress the *PtMAPKK4* gene in tobacco, we selected the vector pCXSN with strong promoter of 35S for vector construction (Figure 4A). The *PtMAPKK4* gene was amplified from the genomic DNA of *Populus trichocarpa* (Figure 4B) using gene-specific primers (Table 1). The recombinant vector *pCXSN-PtMAPKK4* was transformed into *Agrobacterium tumefaciens* EHA105 strain (Figure 4C). The target gene was incorporated into tobacco genome using *Agrobacterium-*mediated genetic transformation. Five transgenic lines were validated by PCR (Figure 5). Northern blot analysis on RNA extracted from leaves showed high transcript levels in transgenic lines but none in WT at target situation. A lower intensity hybridization signal detected in the WT lane might be caused by homologous gene fragment of the probe in tobacco. The band detected under the target band was caused by RNA fracture (Figure 6). The expression levels of #2, #4, and #5 were higher than those of #1 and #3. Transgenic lines were cultured to harvest T2 homozygous seeds for an analysis of salt stress resistance.

**Figure 7:**
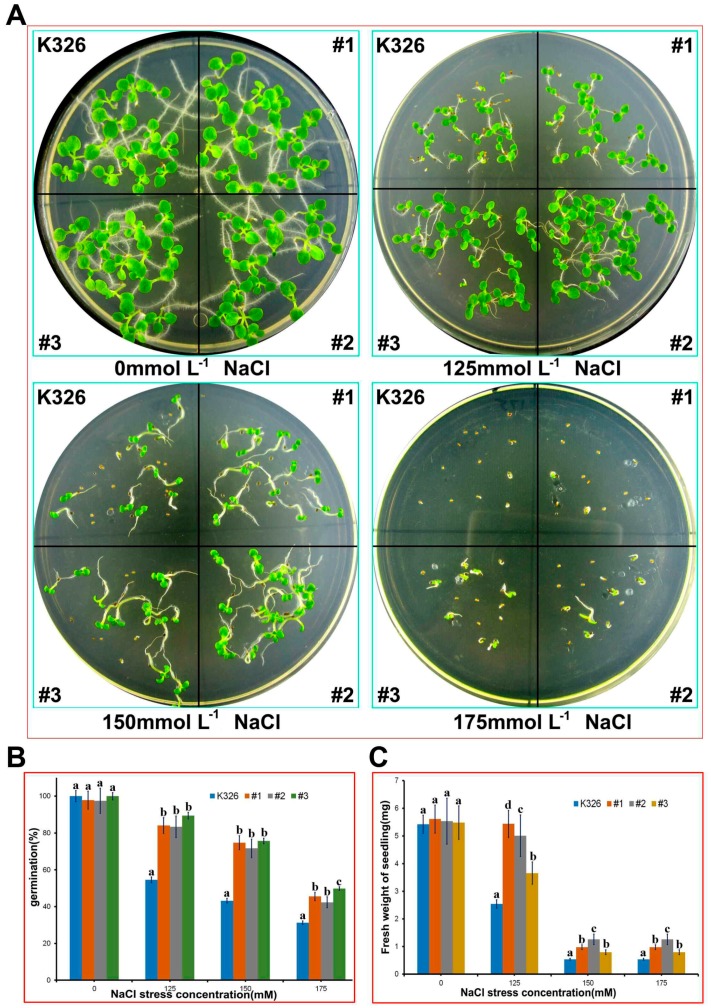
Resistance analysis of *PtMAPKK4* transgenic tobacco and WT in 0, 125, 150, and 175 mmol·L^−1^ NaCl culture at germination stage (**A**); Germination rate (**B**) and fresh weight (**C**) were measured separately. Letters a, b, c and d represent a significant difference of the means at *p* < 0.05.

The #1, #2, and #3 homozygous lines were selected for a salt tolerance experiment. Seeds of transgenic and WT were germinated on medium containing different salt concentrations 0 (control), 125, 150, and 175 mM NaCl for 14 days. Under the control condition (in the absence of NaCl), the WT and transgenic lines showed no significant difference in germination rate and fresh weight. However, in the presence of NaCl (125–175 mM), the germination rate and fresh weight were statistically higher in the transgenic lines than in the WT (Figure 7). The improved germination rate and growth under NaCl stress in the transgenic lines suggests a contribution of PtMAPKK4 to biological function of plant tolerance to NaCl stress. Overexpressed lines were better than WT with regard to growth status or germination rate in each NaCl stress concentration, that is, the *PtMAPKK4* gene contributed to the biological function of plant tolerance to NaCl stress.

**Figure 8:**
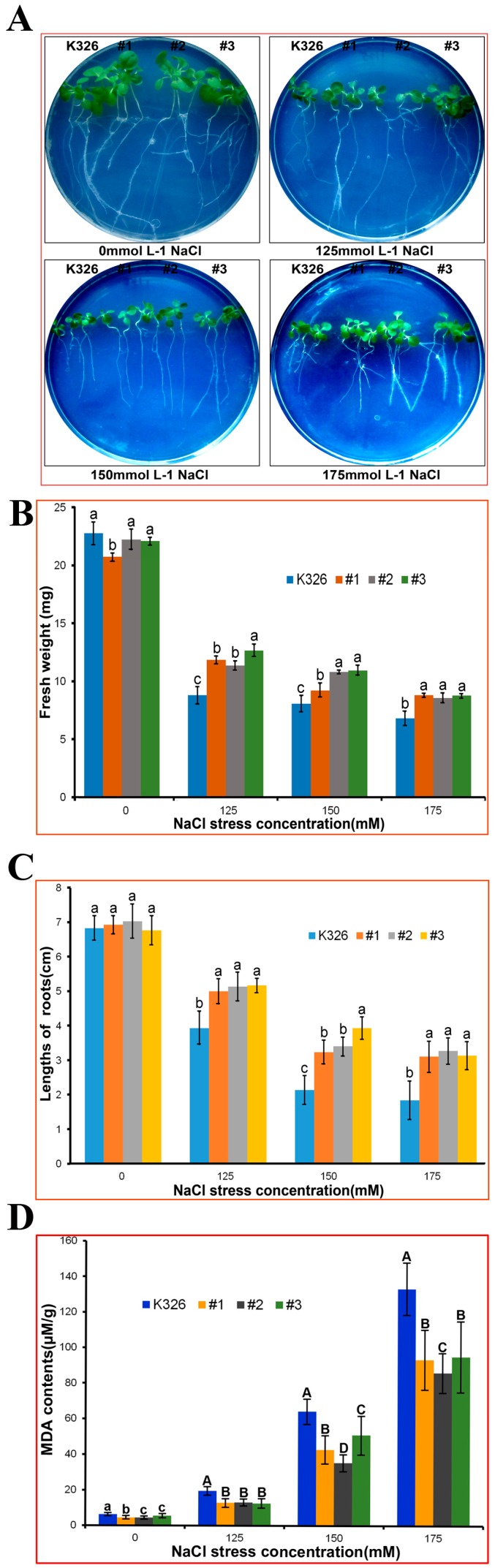
Resistance analysis of transgenic tobacco and WT in 0, 125, 150, and 175 NaCl culture after germination for 9 days (**A**). Fresh weight (**B**); lengths of roots (**C**); and MDA contents (**D**) were measured separately. Differences were analyzed by ANOVA (*p* <0.05).

Seeds of #1, #2, and #3 transgenic homozygous lines and WT were germinated and grown on 1/2 MS medium for 9 days. Consistently grown seedlings were selected for transplant onto 1/2 MS medium containing 0 (control), 125, 150, and 175 mM NaCl and were vertically placed on the dish for 15 days. Growth of seedlings in all salt treatment groups was significantly inhibited by salt. However, transgenic lines showed better salt tolerance than WT (Figure 8A). Root length and fresh weight of transgenic lines were higher than those of WT (Figure 8B,C). MDA content serves as an indicator of plant cell oxidative damage; NaCl stress treatment increases MDA content in plants. This study detected MDA content of WT and overexpressed lines under different treatments (Figure 8D). MDA content was low and not significantly different between WT and overexpressed lines in 0 mM NaCl control. However, MDA content positively correlated with salt concentration in treatment groups and was significantly higher in WT than in overexpressed lines. Overexpression of *PtMAPKK4* alleviated salt stress-initiated oxidative toxicity of plant cells.

## 3. Discussion

MAPKK plays a significant role in responses to environmental stresses during plant growth and development [24]. MAPKK takes part in the regulation of downstream target genes through signal components for signal amplification; the amplification of a cascade reaction regulates the adaptive growth of plant cells [25]. The subcellular localization of proteins is valuable indirect evidence for protein function, and provides a preliminary cue for elucidating protein function [26]. Proteins are synthesized in cytoplasm and then transferred to specific organelles by protein-sorting signals [27]; some proteins are secreted out of cells or remain in the cytoplasm. A deviation in protein localization features a profound effect on cell function [28]. Protein presents complex structures and includes various kinds; each protein exhibits its own biological function and is a substance expressing biological properties. However, proteins synthesized inside cells can function only when transported into sorted organelles. Hence, the functions of proteins are associated with their subcellular localization. The prediction of a protein’s subcellular localization features biological significance. Proteins with nucleus localization signals: -Pro-Pro-Lys-Lys-Lys-Arg-Lys-Val- and proteins with nucleus export signals: -Leu-Ala-Leu-Lys-Leu-Ala-Gly-Leu-Asp-Ile- have been previously reported [29]. In this study, the protein subcellular localization PSORT software was used to predict the PtMAPKK4 protein’s subcellular localization. The probability of amino acids containing a nuclear protein sorting signal reached 70%. We constructed the yeast expression vector pYES2-PtMAPKK4-GFP with fusion of GFP. Plasmid DNA was transformed into the yeast strain INVScI. As observed with confocal laser scanning microscopy, fusion protein was only expressed in a yeast’s nucleus (Figure 3).

Studies have shown that in MAPK cascades, MAPKK determines the diversity of signal transduction processes, and MAPKK is induced by abiotic stresses such as salt, oxidation, cold and drought [8,21,30]. Gao et al. discovered that MAPKK, which was encoded by the *Arabidopsis thaliana* At1g73660 gene, down-regulated salt tolerance in this organism [31]. Zhang et al. separated five up-regulated cDNA for genes up-regulated under salt stress through a suppression of subtractive hybridization [32]. Alkayal et al. observed a 2.7% up regulated expression of sequence tag-encoding proteins in the cDNA library of *Dunaliella salina* [33]; most were members of the calcium-dependent signal transduction pathway and the MAPK signal transduction pathway and are related to intracellular communication. The MAPK signal transduction pathway and MAPKK gene play important roles in salt tolerance mechanism of *Dunaliella salina* [34]. Quantitative real-time PCR showed that rice OsMAPKK4 and OsMAPKK6 were induced by the salt stress and cold stress [35]. *Arabidopsis thaliana* MAPKK9 regulates ethylene synthesis through MAPKK9-MAPK3/MAPK6 cascade reaction, and enhances the sensitivity of seedlings to salt [36]. Nakagami et al. proved that AtMAPK4 and AtMAPK6 were involved in the regulation of At MAPKK2 under salt stress, that AtMAPK4 and AtMAPK6 were constitutively activated in AtMAPKK2-over-expressing transgenic plants with the enhancement of salt tolerance, and that in AtMAPKK2 deficient mutants, the activation of AtMAPK4 and AtMAPK6 was blocked, which showed the super salt-sensitivity [21]. These studies indicated that the regulation of MAPKKs and MAPKs under salt stress which improved MAPK cascade reactions, could enhance the salt tolerance of the plant.

In this study, leaves and roots showed significantly higher expressions of *PtMAPKK4* than stems. Expression in leaves increased in response to NaCl stress. In roots, the mRNA level first decreased and then increased. No remarkable stress response was observed in stems. PtMAPKK4-overexpressed tobacco lines showed higher root length and biomass than K326 and fresh weight than WT tobaccos. The MDA concentration in the transgenic lines was significantly lower than that in K326. These results verified that the expression of PtMAPKK4 had effects on plant growth under salt stress. Further studies will focus on signal transduction pathways with the involvement of PtMAPKK4 and its interaction components.

## 4. Materials and Methods

### 4.1. Plant Materials

One-year-old potted *Populus trichocarpa* plants were harvested, and the leaves, stems, and roots were separated and placed in liquid nitrogen for subsequent extraction of total RNA and gene cloning. One-year-old branches of potted *Populus trichocarpa* were cultured for 4 weeks by using hydroponics. The plants were subjected to 150 mM NaCl stress at different time periods, namely, 0 (added water as control), 6, 12, 24, and 48 h. Leaves, stems, and roots were separated and frozen in liquid nitrogen for analysis of a *PtMAPKK4* expression pattern.

*Nicotania tabacum* (K326) seeds were provided by the Agricultural College of Yanbian University. Tobacco seeds were disinfected by 75% alcohol and sterilized by 1% NaClO after washing with sterile water. Seeds were germinated on 1/2 Murashige–Skoog (MS) medium after rewashing. Finally, aseptic seedlings were obtained.

### 4.2. RNA Extraction and Preparation of cDNA

Briefly, 0.5 g of each sample was separated for total RNA extraction by using TRIzol reagent (Invitrogen, Carlsbad, State of California, America). After obtaining total RNA, 1 µg of total RNA was used for each transcriptase reaction with reverse transcriptase kit (TOYOBO, Osaka, Japan) to produce cDNA.

### 4.3. Gene Cloning and Plasmid Constructions

Total RNA were extracted from the leaves, stems, and roots of *Populus trichocarpa* and were used to produce cDNA. We obtained the nucleotide sequence of PtMAPKK4 and designed primers (Table 1) for cloning according to the accession number of PtMAPKK4 in Genebank, XM_002315352 (*Populus trichocarpa* MAP KINASE KINASE 4 family protein, POPTR_0010s25570.1.p, Gene. PtMAPKK4). The high-fidelity thermostable DNA polymerase EX-taq (Takara, Osaka, Japan) was used for the amplification of targeted fragments, which were connected with the cloning vector pMD18-T and transformed into *Escherichia coli* JM109 (Transgen Biotech, Beijing, China). Plasmids were extracted for verification through double-enzyme digestion with EcoR I and Hind III (Fermentas, Vilnius, Lithuania) and were subsequently sequenced.

Using the pMD18-T-PtMAPKK4 plasmid as template, primers containing restriction sites were used for PCR amplification (Table 1). The targeted fragments were connected with pMD18-T and sequenced. pBS-MCS-GFP and the recombinant plasmid pMD18-T-PtMAPKK4 underwent double-enzyme digestion using Kpn I and Spe I. The recombinant plasmid pBS-*PtMAPKK4-GFP* was constructed after gel extraction and purification. The yeast expression vector pYES2 and the recombinant vector pBS-*PtMAPKK4-GFP* were both digested using Kpn I and EcoR I. After digestion, fragments of pYES2 and pBS-*PtMAPKK4-GFP* were extracted for ligation using T4 ligase (Fermentas, Vilnius, Lithuania). The recombinant plasmid was transformed into JM109 and sequenced. pYES2*-PtMAPKK4-GFP* and pYES2*-GFP*(contrast) were used for the subcellular localization of proteins.

Using the recombinant vector pBS-*PtMAPKK4-GFP* as template, targeted fragments were amplified by PCR using gene-specific primers (Table 1). pCXSN plasmids were digested by Xcm I and subsequently ligated with target fragments. The recombinant plasmid pCXSN-*PtMAPKK4* was transformed into JM109 and sequenced. The vector pCXSN*-PtMAPKK4* was transformed into *Agrobacterium tumefaciens* EHA105 for tobacco genetic transformation. 

### 4.4. Bioinformation Analysis

The open reading frame (ORF) and amino acid sequences of *PtMAPKK4* were obtained from the National Center for Biotechnology Information (NCBI) (https://www.ncbi.nlm.nih.gov). Three-deimensional (3D) protein structures were predicted online. We analyzed the structures of amino acids coded by the target gene, homologies, isoelectric points, and molecular weights. MAPKK members of *Arabidopsis thaliana*, *Tarenaya hassleriana*, *Gossypium raimondii*, *Citrussinensis*, *Betavulgaris*, *Pyrus bretschneideri*, *Fragaria vesca*, *Vitis vinifera*, and *Populus triocarpa* were used to create a phylogenetic tree with DNAMAN software. The subcellular localization of obtained PtMAPKK4 protein was predicted by PSORT (http://psort.hgc.jp/form2.html). Conserved domains of protein were analyzed by SMART (http://smart.embl-heidelberg.de/smart/set_mode.cgi?NORMAL=1).

### 4.5. qRT-PCR Analysis

cDNA was diluted 10 times and was used as template for qRT-PCR. qRT-PCR assays were performed using SYBR Green QPCR Master Mix (TOYOBO, Osaka, Japan). Tobacco *Actin2* was used as housekeeping gene. Primers were designed by Primer5.0 (as shown in Table 1).

### 4.6. Subcellular Localization Analysis

Recombinant vector was chemically transformed into yeast strain INVScI (Invitrogen, Carlsbad, CA, America) by induction with polyethylene glycol/lithium acetate. Transformed yeast strains were screened on SD/-Ura medium for 2–3 days under 30 °C until monoclone development. Transformed monoclonal yeast strain spots were selected and cultured in 5 mL of yeast extract peptone dextrose medium overnight at 30 °C. Briefly, 100 µL of bacterio-liquid was centrifuged and washed twice with sterile water. Afterward, 300 µL of yeast extract-peptone-glycerol (YPG) was added to resuspend yeast cells. Expression of recombinant plasmids was induced by placing cells in shaking flask containing 2.0 mL of YPG medium under shaking cultivation at 30 °C for 4 h. Any localization of PtMAPKK4-GFP in yeast cells was observed by using confocal laser scanning microscopy per 1 h.

### 4.7. RNA Gel Blot Assay

Total RNA was obtained using TRIzol. Denaturing gel electrophoresis was conducted with RNA gel blot analysis according to Sambrook et al. to examine transformation of *PtMAPKK4* gene, which was marked with a *digoxigenin* probe [37].

### 4.8. Plant Genetic Transformation and Analysis of Salt Tolerance

T-DNA region of recombinant vector pCXSN*-PtMAPKK4* was inserted into tobacco genome through *Agrobacterium*-mediated method. Transgenic lines were identified through RT-PCR by using gene-specific primers. T2 generation homozygous seeds were harvested for analysis of salt tolerance.

Seeds of T2 transgenic lines (*PtMAPKK4*: T2-#1, #2, #3) and wild-type (WT) (K326) were disinfected by alcohol and sodium hypochlorite. Twenty-five grains of each line were selected and sowed on 1/2 Murashige and Skoog (MS) + 0, 125, 150, and 175 mM NaCl media. 1/2 MS medium was used, and experiments were repeated thrice. Seeds were vernalized in incubators for 7 days at 4 °C. Statistical results were recorded at the beginning of the 7th day until the 14th day, and total germination rate was calculated. Investigations determined growth phenotype, fresh weight, and differences in malondialdehyde (MDA) contents between samples.

Seeds of T2 transgenic lines (*PtMAPKK4*: T2-#1, #2, #3) and WT (K326) were disinfected and sowed on 1/2 MS medium. Seeds were vernalized for 7 days at 4 °C and transported to incubators. On the 9th day after germination, seedlings with similar growth to that of WT were selected and placed in 1/2 MS + 0, 125, 150, and 175 mM NaCl media under 2600LX illuminance and 16/8 h (light/dark) cycle. Growth conditions were analyzed after 15 days. Differences in fresh weight and statistical data were calculated using Excel to determine phenotype changes and to detect effects of NaCl stress on root lengths in each line.

### 4.9. Statistical Analysis

All experiments were performed in triplicate. Values in figures and tables represent mean ± standard error (SE) of three independent experiments (*n* = 3). Differences were analyzed by ANOVA (SPSS18.0 program). Different letters indicate significant differences of means between treatments (*p* < 0.05).

## Figures and Tables

**Table 1:** Primers used for quantitative RT-PCR (qRT-PCR) and gene cloning.

Primer	Primer Sequence
Actin2-F	5′-TTCTACAAGTGCTTTGATGGTGAGTTC-3′
Actin2-R	5′- CTATTCGATACATAGAAGATCAGAATGTTC-3′
*PtMAPKK4*-Q-F	5′-GCCATCGCCATAGTAGTTCTCCT-3′
*PtMAPKK4*-Q-R	5′-CATAACCCAAGGCTGTGGAACT-3′
*PtMAPKK4*-1F	5′-CACCGAGTCATGAAACCAGTTC-3′
*PtMAPKK4*-1R	5′-ACCCACTAAGGAATTGGCCTAG-3′
pYES2-*PutMAPK4*-G-F	5′-GGTACCATGAAACCAGTTCAACC-3′
pYES2-*PutMAPK4*-G-R	5′-ACTAGTAGGAATTGGCCTAG-3′
pCXSN*-PutMAPK4-*F	5′-CACCGAGTCATGAAACCAGTTC-3′
pCXSN*-PutMAPK4-*R	5′-ACCCACTAAGGAATTGGCCTAG-3′
